# *Mate Yako Afya Yako*: Formative research to develop the Tanzania HIV self-testing education and promotion (Tanzania STEP) project for men

**DOI:** 10.1371/journal.pone.0202521

**Published:** 2018-08-27

**Authors:** Donaldson F. Conserve, Kathryn E. Muessig, Leonard L. Maboko, Sylvia Shirima, Mrema N. Kilonzo, Suzanne Maman, Lusajo Kajula

**Affiliations:** 1 Department of Health Promotion, Education, and Behavior, Arnold School of Public Health, University of South Carolina, Columbia, South Carolina, United States of America; 2 Department of Health Behavior, Gillings School of Global Public Health, University of North Carolina at Chapel Hill, Chapel Hill, North Carolina, United States of America; 3 Tanzania Commission for AIDS (TACAIDS), Dar es Salaam, Tanzania; 4 Department of Psychiatry and Mental Health, Muhimbili University of Health and Allied Sciences, Dar es Salaam, Tanzania; University of California, UNITED STATES

## Abstract

The purpose of this formative research, guided by the Integrated Behavioral Model, was to assess men’s attitudes and personal agency towards HIV self-testing (HIVST) and confirmatory HIV testing in order to inform the development of the Tanzania STEP (**S**elf-Testing Education and Promotion) Project, a peer-based HIV self-testing intervention for young men in Tanzania. Qualitative in-depth interviews were conducted with 23 men in Dar es Salaam, Tanzania who socialize in networks locally referred to as “camps”. Men reported privacy, confidentiality, and saving time as the primary reasons for their self-testing interest. Most participants had high perceived control and self-efficacy to self-test and seek confirmatory HIV testing. Nevertheless, men reported concerns related to their ability to perform the test and the potential lack of post-test counseling. Specific recommendations for the intervention included providing HIVST education and pre-test counseling, and using mobile health (mHealth) strategies for participants to reach a healthcare professional for further assistance. The findings suggest that while HIVST is highly acceptable among men in Tanzania, future interventions will need to address the challenges that men may face with HIVST before promoting it as an alternative or supplement to facility-based HIV testing.

## Introduction

In 2015, an estimated 36.7 million people were living with HIV globally and nearly 40% of people living with HIV (PLWH) were unaware of their HIV status [1, 2]. In an effort to end the HIV/AIDS epidemic, the Joint United Nations Program on HIV/AIDS (UNAIDS) launched the “90-90-90” targets, with the first two goals focusing on achieving that 90% of PLWH learn their status and 90% of those diagnosed with HIV are linked to antiretroviral treatment (ART) [3]. HIV testing is crucial for achieving these targets because it is the first step in learning one’s HIV status and initiating ART for people unaware of their positive HIV status [4]. Though HIV testing services have expanded in the past decade, HIV testing uptake continues to remain lower among men compared to women [4]. The lower HIV testing uptake has resulted in fewer men than women learning their HIV-positive status for the first 90 target in Tanzania. According to the 2016–2017 Tanzania HIV Impact Survey (THIS), only 45% of men living with HIV were aware of their status compared to 56% of women [5]. Among men living with HIV who were aware of their status, 86% of them reported use of ART and 84% of those using ART were virally suppressed [5]. These data suggest that once men who are living with HIV learn their status, a high number of them initiate ART and reach viral suppression, thereby supporting the need to increase HIV testing among men in order to facilitate the achievement of the 90-90-90 targets among men and reduce HIV transmission.

As a response to the high rate of men living with HIV who are unaware of their HIV status, the Tanzania Commission for AIDS (TACAIDS) combined efforts with the National AIDS Control Programme (NACP), USAID, FHI 360 and implementing partners to launch a national Test and Treat Campaign on June 19^th^, 2018 called *Furaha Yangu*! (*My Happiness*!), with a focus on engaging adult men and adolescent boys in HIV counseling, testing, and treatment [6–8]. As part of this effort, TACAIDS, UNAIDS Country Team, Benjamin Mkapa Foundation (BMF) and other partners are developing the 2018–2020 Catch-Up Plan to increase HIV services uptake among men and adolescent boys [9]. The plan will provide guidance for the nationwide campaign for men and include a set of intervention activities, including HIV self-testing (HIVST), that will be implemented in the next two years [9]. Given that HIVST will be one of the national strategies to increase HIV services uptake among men, the project described in this paper will inform the country’s efforts by identifying potential barriers and enablers of HIVST for men. HIVST, which allows a person to collect their own specimen and perform their HIV test in private, may work better for men who are not reached by facility-based testing [10–12]. In 2016, the World Health Organization (WHO) released guidelines for HIVST and recommended it as a complementary HIV testing approach to reach people (i.e. men) who are not accessing current testing services [13]. Following the WHO HIVST guidelines release, the number of countries adopting HIVST in their national policies increased from 16 to 40 [14].

As of July 2018, Tanzania’s National HIV Testing Guidelines do not allow HIVST [15]. However, there are plans to change the law by the end of 2018 to allow HIVST [16]. In addition, NACP and implementing partners began piloting HIVST projects focusing on key populations to inform the national policy and implementation of HIVST in the country [9]. Since HIVST is not yet allowed in the country there is little information about HIVST acceptability and feasibility, with the exception of two studies reporting that 66% of men in Dar es Salaam who had not tested for HIV in the past 12 months were willing to self-test and that men perceived several advantages and disadvantages to HIVST [17, 18]. Research conducted in other countries in sub-Saharan Africa suggests that HIVST is highly acceptable [19] and reached a high proportion of men in Malawi, with 90% of men aged 16–19 years old reported to have self-tested in 14 neighborhoods where trained resident volunteer-counselors offered oral HIVST kits [20]. In Kenya, secondary distribution of HIVST kits by female sex workers and women receiving antenatal and post-partum care to their male partners was also acceptable and feasible [21, 22]. While these studies have provided preliminary evidence of HIVST acceptability and different strategies to distribute HIVST among men, further research is needed to examine men’s perceptions towards HIVST and follow-up confirmatory HIV testing in case of a positive result in order to inform the successful implementation of HIVST for men.

Based on the Integrated Behavior Model (IBM), a person’s intention to perform a behavior (e.g. HIV self-testing and seeking confirmatory HIV testing), is influenced by their attitude (experiential and instrumental) toward the behavior, perceived norm (injunctive and descriptive), and personal agency (self-efficacy and perceived control) [23]. *Experiential attitude* is defined as the emotional response to the idea of engaging in a behavior whereas *instrumental attitude* refers to the beliefs about the outcomes of the behavior [23]. *Injunctive norm* relates to the normative beliefs about what others in the person’s social network think one should do regarding the recommended behavior and *descriptive norm* results from an individual’s perception of whether people in their network engage in the recommended behavior [23]. *Perceived control* describes to what extent a person perceives that their environment facilitates or impedes them from performing the behavior and *self-efficacy* is the amount of confidence a person has in their ability to perform the behavior [23]. Prior quantitative research, conducted by our team, among men in Tanzania revealed that descriptive norms were associated with previous HIV testing while willingness to self-test for HIV in the future was correlated with injunctive norms [17, 24]. Identifying how other IBM constructs (i.e. attitudes and personal agency) are related to men’s willingness to self-test for HIV and, if needed, to seek confirmatory HIV testing is important to design effective HIVST interventions for men.

For this purpose, we employed two (attitudes and personal agency) of the IBM constructs to assess qualitatively men’s attitudes and personal agency towards HIVST and confirmatory HIV testing to initiate care and treatment. In addition, we inquired about recommendations to address perceived challenges related to HIVST and seeking confirmatory HIV testing in order to inform the development of the Tanzania STEP (**S**elf-**T**esting **E**ducation and **P**romotion) Project [25] (locally referred to as *Mate Yako Afya Yako (Your Saliva Your Health)*.

## Methods

### Study setting and recruitment

The data were collected within four wards (Mabibo, Manzese, Tandale, Mwanyanamala) of Kinondoni Municipality in Dar es Salaam, Tanzania where HIV testing services are provided for free [26]. The participants were recruited from stable social networks locally referred to as ‘camps’[27]. The camps were first identified as part of the preliminary research activities conducted for a randomized controlled trial (RCT) designed to assess the efficacy of a microfinance and health leadership intervention to prevent HIV and gender-based violence among camp members (5 R01 MH098690) [27, 28]. More details are provided about the selection and assignment of the camps for the RCT in prior publications [27, 29]. Briefly, camps are social gathering places where networks of mostly young men frequent with elected leaders and dues paying members [27]. Camp members were invited to participate in this formative research sub-study through follow-up phone calls using the phone numbers collected during the baseline and midpoint assessments for the RCT. Participants were purposively recruited based on their responses to the quantitative midpoint survey in which participants were asked about their HIV testing history, HIVST awareness, prior HIVST use, and willingness to self-test for HIV in the future. In order to have a broad range of views, we sampled four sub-groups of men. The sample included men who reported in the midpoint survey that they: 1) had been tested for HIV and were willing to self-test; 2) had been tested for HIV and were not willing to self-test; 3) had not been tested for HIV and were willing to self-test; and 4) had not been tested for HIV and were not willing to self-test.

### Data collection

From November to December 2015, 23 participants were interviewed using a semi-structured guide. Although we intended to interview more men, we ended with 23 because we reached saturation due to the similarity in the information the participants were sharing. Informed by the IBM, we asked questions to assess men’s attitudes and personal agency towards HIVST and confirmatory HIV testing. Sample interview guide questions included: *Can you tell me some of the reasons that would or would not interest you to try HIV self-testing*? *What do you think the challenges*, *if any*, *would be for you to use a self-testing kit*? *What can be done*, *if anything*, *to remove the challenges to you using an HIV self-test kit*? *How*, *if at all*, *could we encourage someone who self-tested to go for a confirmatory test at the clinic*? The interviews were conducted by two college-educated male Tanzanians trained in qualitative research methods and on HIVST. Interviewers were provided with HIVST kits and a video about HIVST in Kiswahili to show participants before the interview in order to familiarize them with HIVST. Each interview was conducted in Kiswahili, the national language in Tanzania, and lasted between 30–60 minutes. Participants received 10,000 Tanzanian shillings (TSH), approximately $ 4.50, as compensation for their study participation. In addition to the individual interviews, a community information session was held on February 16^th^, 2018 in Manzese and Tandale, the sites for the STEP Project, to share the formative research findings with 52 community members, including 19 camp leaders, 6 camp guardians, 2 ward executive officers, 14 municipal executive officers, 2 ward health officers, 2, VCT focal persons, 2 Care and Treatment focal persons, 2 youth friendly services focal persons, 2 District AIDS Coordinator, and 1 non-governmental organization member. During the meeting, the STEP Project was introduced to community members and information about HIVST was provided. Community members also had a chance to share additional recommendations and develop a Swahili name for the STEP Project. They suggested three names and eventually agreed that one of these names—*Mate Yako Afya Yako (Your Saliva Your Health)* captured the objectives of the study given the use of saliva for the self-testing process and believed that this name would catch people’s attention. The views and recommendations shared during the community information session were similar to those reported in the individual interviews, which are the focus of this paper.

### Ethical approval

The study was approved by the University of North Carolina at Chapel Hill Institutional Review Board and the Muhimbili University of Health and Allied Sciences Senate Research and Publications Committee. Written informed consent was obtained from all participants prior to data collection.

### Data analysis

Interviews were audio recorded, transcribed, and then translated into English for analysis. The analysis focused on the following *a priori* codes: attitudes (experiential and instrumental) and personal agency (self-efficacy and perceived control) towards HIVST and confirmatory HIV testing as well as recommendations for a future HIVST intervention. Prior to the analysis, experienced qualitative research team members provided training in qualitative data analysis to less experienced members (undergraduate research assistants). The research team developed a formal codebook for the deductive codes; this document contained a description and example of each code [30]. We coded the transcripts in analytical phases by applying deductive codes but also identifying emerging codes in the second phase of analysis. Initially, 3 sub-teams of six coders reviewed the data and applied these structural (deductive) codes based on the interview questions and probes. Qualitative analysis software, ATLAS.ti 7.5, was used for coding and generating code reports. To ensure reliability and validity, each interview was coded independently by at least two research team members [31]; each sub-team coded a subset of data and held a consensus-coding meeting to compare their respective application of codes and resolve any discrepancies. A code summary (with quotations) was created for each code and used in the second phase of the analysis. In that phase, the senior coders coded data to generate more specific subtopics addressing the deductive codes. These codes were based on identifying patterns of responses across data and addressing the dimensions of the broader deductive codes. The research team developed and refined a formal codebook, which contained a description and example of each code [30]. Analytic rigor was ensured through researcher triangulation. We met regularly to establish agreement regarding code definitions, code application, and selecting quotations for illustrative purposes.

## Results

### Participants characteristics

As shown in Table 1, the mean age was 27.3 years (± 6.5), ranging from 20 to 51 years old. Less than half (39%) of the men had obtained a secondary education. Nearly half of the men were single (n = 11, 48%), married or cohabiting (n = 11, 48%) as compared to having a non-cohabiting partner (n = 1, 4%). Fifteen (65%) of them were self-employed and approximately half (n = 12, 52%) had obtained an HIV test in the past 12 months. Most men (n = 18, 78%) had no prior knowledge of HIVST but willingness to self-test was relatively high (n = 15, 65%).

**Table 1:** Participant demographic characteristics.

	Mean (SD) (Min -Max)	Frequency	%
Age	27.3 (+6.5) (20–51)		
Education			
No formal education		1	4
Primary		12	52
Secondary		9	39
Higher than secondary		1	4
Marital status			
Single		11	48
Married/cohabiting		11	48
Girlfriend		1	4
Employment			
Employed		2	9
Self-employed		15	65
Unemployed student		1	4
Unemployed non-student		5	22
HIV test in the past 12 months			
No		10	52
Yes		12	48
Heard of HIV self-test			
No		18	78
Yes		5	22
Willing to self-test			
No		8	35
Yes		15	65

### Attitudes toward HIVST and confirmatory HIV testing

As previously described, experiential attitude (feelings about a behavior) and instrumental attitude (beliefs about the outcome of a behavior) influence the intention to perform a new behavior. In this section, we present the experiential and instrumental attitudes that the participants reported towards HIVST and seeking confirmatory HIV testing. Though most participants were unaware of HIVST, they had a positive emotional response (experiential attitude) towards HIVST after watching the video and they were willing to self-test in the future. The positive emotional response was illustrated in the following quote: “*I will feel happy because even if you discover that you have got problems (HIV) it becomes your own secret unlike going to the clinic as you will meet different people*.*”* (Single, 28 years old, and not tested)

One participant who described being afraid of HIV testing in general, reported a negative experiential attitude toward both hospital-based testing and HIVST. He stated, “*Yes*, *it is scary at the hospital in comparison to home*. *I took a friend of mine to Magomeni (hospital) for a HIV test and I let him/her go in while I wait outside…Even though I am testing myself privately (HIVST) but still I am scared you have it in your living room*…*Well when you see how others suffer that gives me a headache*. (Single, 26 years old, and not tested)

Regarding the need to seek confirmatory HIV testing in case of a positive or inconclusive self-test result, most participants reported positive experiential (feelings about a behavior) and instrumental (beliefs about the outcome of a behavior) attitudes and mentioned the ability to receive treatment and governmental assistance as two motivators to attend the clinic to verify one’s positive self-test result. The following quotes demonstrate the positive experiential and instrumental attitudes toward seeking confirmatory HIV testing:

*I will go to the clinic for counselling and medicines because after testing and finding that you are HIV-positive you will be using medicines (ARV)… Those of reducing the intensity of the virus*. (Single, 21 years old, and not tested)

*The assistance there is that after you have already known your results you have to go to report at the health centre so that you start using medicines*, *and when you go there it means that you will come to get other assistance from the government*. (Married, 36 years old, and tested)

### HIVST benefits: Privacy, removal pre-test counseling anxiety, time saving

Participants reported positive instrumental attitude (beliefs about the outcome of a behavior) about HIVST that would motivate them to self-test in the future. The positive instrumental attitude related to the benefits HIVST would afford them such as the ability to test in private compared to testing at a healthcare facility. The privacy would encourage men who are afraid of being seen at a clinic or meeting a counselor at the clinic from their neighborhood, leading to a potential breach of confidentiality and community stigma if a person is infected. Two participants described the following:

*The main thing is that you can go to test at the health centre but we live with the same health providers in the streets so you can test but after two minutes*, *you start getting fingers pointing at you on the ways and the issues of stigmatization*. *But with self-testing you test yourself provided you have got the instructions to use that instrument and test to know your health and how should you do or live*. (Cohabitating, 28 years old, and not tested)

*It will give people privacy as you can take it and go test anywhere in privacy as most of the time people fear going to health clinics as they may meet someone they know or they know a worker there who after testing might go spread the results*. (Cohabitating, 22 years old, and tested)

In addition to the privacy associated with self-testing, another perceived benefit of HIVST that men reported was the minimization of the anxiety and fear that pre-test counseling at the clinic and hospital create. The following quotes illustrate this point:

*In the beginning I said that I had fear so if an instrument like this will be available I will test it myself and it will be my secret unlike going to test at the hospital as going there to test for HIV you will first meet with counselling and that fear is the thing which I don’t want*. (Single, 21 years old, and not tested)

*Well I think the fear subsides because when you are at the clinic sometimes the advice from counsellors are a bit scary like what will you do if you are positive and the like*. *Those words are the ones scaring people*. (Married, 31 years old, and HIV tested)

The other benefits of HIVST that were reported were that it would provide an alternative to repeat testing and save time as described by one participant:

*There was a time you have to go to specific places and you cannot say if you test once that is it*, *you have to stay for three months and test again*. *Now you can go to a center to test for the first time and the rest of the times you use the self-test kit*. *So it saves time and it is easier*…*So for 20 minutes*, *and then I have other hours to do different activities around home without wasting much more time to go to the clinic and do the test*, *so it becomes really easy*. (Cohabitating, 28 years old, not tested)

### Disadvantages of HIVST: Possible harm due to lack of counseling

Participants also reported negative experiential (feelings about a behavior) and instrumental (beliefs about the outcome of a behavior) attitudes towards HIVST. The main concern was the lack of post-test guidance associated with self-testing compared to testing at the hospital, which may lead to individuals harming themselves in case of a positive self-test result. The following quotes illustrate these concerns:

*I like in the hospital because it’s where you can get good instructions*. *For example*, *if you are found positive you can be told that do this and this and this unlike self-testing because you can test yourself and know that truly you are positive but fail to know which directives to follow so that you can live in hope*. (Single, 23 years old, and not tested)

*Self-testing needs confidence and willingness*. *One may decide to go for a test and when things turn out bad they may become irrational and commit suicide like that is the best decision of all while the problem is they did not receive proper counselling prior the test*. *And there are medications that one can use to help them live longer and healthier and constantly following doctor’s advice*. (Single, 24 years old, and not tested)

### Personal agency toward HIVST and confirmatory HIV testing

As mentioned above, personal agency refers to the *perceived control* (environment facilitates or impedes behavior) and *self-efficacy* (confidence a person has in their ability to perform the behavior). Many participants reported an increase in their ability to use the HIVST kit correctly (self-efficacy) after viewing the video. One participant stated, “*Testing myself accurately is possible although I will have to see the testing procedure three or four times so as to be able to test myself correctly*. (Cohabitating, 22 years old, and tested)

Another participant further explained in his response that people should self-test in the morning in order to make it easier to follow all the self-testing steps: *I can because I have understood the video… If you eat*, *brush your teeth that also can be a problem*, *so maybe testing should be the first thing one does in the morning before doing anything*. (Single, 23 years old, and not tested)

However, a few participants expressed low self-efficacy to use the HIVST correctly and were hesitant to self-test because of potential mistakes. They shared that these mistakes and a lack of confidence in self-testing compared to testing at the hospital would reduce their trust in their self-test results. The following quote captured this concern:

*That is the challenge which I will get as I will not be confident as if I will go to the hospital*… *I mean I will not be more confident that the instrument has shown correctly the results*… *Perhaps there may be a certain mistake which I have made or there may be something which I have done wrong*…*Because I may do it wrong and it shows me that I am HIV negative while I am HIV positive*, *so don’t trust myself*. (Married, 28 years old, and tested).

Similarly, participants reported a mixed level of perceived control and self-efficacy to seeking confirmatory HIV testing, with most being confident they would if needed. Not surprisingly, the participant who stated his lack of confidence in self-testing compared to the hospital described his readiness to seek confirmatory HIV testing: *Truly if I see two lines have appeared I will be ready to move from this place to the responsible place to verify my results*. (Married, 28 years old, and tested).

However, the same participant who had a general fear for HIV testing at the clinic earlier reported low perceived control (environment facilitates or impedes behavior) toward seeking follow-up HIV services due to the stigma in his community toward people living with HIV. Notably, this participant described his concerns around HIV testing alongside observations of how the illness has affected his mother and the mistreatment HIV-positive people in his neighborhood receive:

*My biological mother who I live with has the illness… Her condition (feeling sad)*, *she itches 24 hours it is scary when I look at her and think about it*, *it hurts me a lot… You look at the others struggling and you wonder what will happen if it were you*? *That is what depresses us most*… *Yes*, *there are things like being discriminated when others realize that you are infected with HIV****…*** *So one thing*, *if I am infected I will not go anywhere for medications I will just sit over it*, *wait for long*…*until my health status weakens that is when I will go for medications*, *that is how it is*. (Cohabitating, 26 years old, and not tested)

### Recommendations for intervention

#### HIVST education

Participants offered several suggestions for strategies to address the perceived challenges for an intervention to promote HIVST and confirmatory HIV testing for linkage to care. Providing education on how to use the kit properly and how to follow the necessary steps after self-testing was one recommendation to help increase HIVST knowledge, which in turn may change participants’ attitudes and personal agency towards HIVST and confirmatory HIV testing. This is explained in the following quote: *First*, *before distributing those test kits there should be a seminar*, *educating each Tanzanian in general for them to know to use the kit and then getting the results and what to do after that*, *this will make someone more aware*. (Single, 23 years old, and not tested)

Different strategies were mentioned by participants to offer HIVST education such as a counsellor in person or via pamphlets, and through media such as radio and newspapers.

*The education can be given through any means there could be a small pamphlet inside the test packet to educate a person before testing or face to face advice from a counsellor before testing*. *Other methods like education via the media as in radios*, *newspaper*. (Cohabitating, 22 years old, and tested)

#### Screening to assess readiness to handle positive self-test result

To mitigate possible self-harm, one participant recommended screening to determine an HIVST user’s mental/emotional preparedness to deal with a positive self-test result.

*If it will not be possible to check pressure then there is another way where you look at how the person is…Through conversation with the client you are needed to look at him and say that from my questions and his responses is he not going to commit suicide if we give him this instrument*. (Cohabitating, 28 years old, and tested)

#### Mobile health (mHealth) post-test counseling

Participants reported that HIVST users could either receive a phone number or leave their phone numbers in order to receive counseling and other assistance using the self-test kits via mHealth strategies such as phone calls and text messages. Without the phone information, some participants reported it would be impossible for a counselor to reach self-testers and determine if they need post-test counseling.

*You know to convince someone is until you get him but did he leave his mobile number when he tested*? *If he left his mobile number you can call him and educate him and that is the only influence*. *But by self-testing inside without leaving your mobile number you cannot convince him because where will you find him as he has already tested alone and found he is positive*. (Single, 31 years old, and not tested)

Another participant mentioned he would take the initiative to notify the healthcare professional about his self-test result and request guidance on how to proceed. He also suggested that a phone call would be better than text messages since the text messages may not be delivered on time due to poor phone signals.

*The benefits will be there because after testing myself I will be able to call the person concerned and will notify him/her my test results if I am infected then he/she will provide me with guidance or proper instructions on what to do*… *Personally I would just call*, *why texting*? *What if it does not reach the person on time*? *Because currently with network problems on the rise*, *it is better one calls*. (Single, 23 years old, and tested)

## Discussion

For the first aim of this study, men’s attitudes and personal agency towards HIVST and seeking follow-up services such as confirmatory HIV testing were assessed. Since Tanzania has not implemented HIVST, many participants were not aware of HIVST, indicating the need for HIVST awareness campaigns to increase HIVST knowledge and ensure successful implementation and scale-up. Despite the lack of HIVST awareness, most participants had favorable experiential and instrumental attitudes towards HIVST, parallel to findings from studies that included men in Zambia, Malawi and South Africa [32–35]. Similarly, men’s attitudes were positive toward confirmatory HIV testing, with many participants citing the availability of lifesaving treatment for HIV as the reason for their interest in seeking follow-up HIV services and care if their self-test results were to be positive. The availability of lifesaving treatment was also found to be an enabling factor for men to access HIV testing services in Kenya and Uganda, with men living with HIV acknowledging the importance of ART in revitalizing their physical and social lives as well as their familial roles [36, 37]. In Tanzania, the NACP provides HIV prevention, care, and treatment [38] and will publish guidelines for how self-testers can receive additional support to confirm their HIV status and start on treatment if needed. HIV treatment has been provided for free in the country since 2004 [38] and 84% of men living with HIV reported to be using ART during the recent 2016–2017 Tanzania HIV Impact Survey [5], indicating that a high proportion of men may seek care and treatment with proper guidance and support after receiving a positive self-test result.

The primary motivators (privacy, confidentiality, and saving time) men reported for desiring to self-test were consistent with other studies [33, 39, 40]. These HIVST benefits are particularly salient for men since our findings revealed that they are reluctant to test at the clinic in fear of confidentiality and privacy breach. Also, the time saving factor of HIVST was appealing to men since long work hours may prevent men from having the time to attend clinics for HIV testing [36, 37]. A study conducted in Uganda found that men spent long hours at the clinics and were sometimes turned away from clinics because of the mismatch between testing hours and work schedules [37]. Another perceived HIVST benefit is the aversion of fear, anxiety, and intimidation associated with pre-test counseling that is mandatory at health facilities prior to HIV testing. Other studies conducted in South Africa have found similar results with people preferring HIVST because they dislike face-to-face counseling and that HIVST would allow them to circumvent the repetitive, frustrating, intrusive, and time-consuming pre-test counseling offered with traditional HIV testing services [34, 41]. In a more recent study, the optional counseling nature of HIVST was also cited as an advantage among people who had tested multiple times and were subjected to the same pre-test counseling repeatedly [34].

Though HIVST was highly acceptable, some participants raised concerns about the potential psychological and physical harm a positive self-test result may lead HIVST users to experience due to the lack of post-test counseling. While these concerns mirror those reported in other formative research conducted in Kenya, Malawi, and South Africa [33, 42], no HIVST-related violence or suicides were found during a two-year community-based HIVST trial in Malawi where 14,004 residents self-tested and 524 individuals were diagnosed with HIV [20]. Similarly, a systematic review of studies conducted to inform WHO guidance found no HIVST-related harm across the trials conducted in Africa [43]. These findings indicate that although interventionists should be mindful about potential HIVST-related harms, the lack of reported HIVST-related suicides and violence suggest that proper HIVST information, pre-test counseling, and facilitated HIV care assessment [20] can prevent HIVST-related harms. Another concern was the mixed finding related to some participants reporting high self-efficacy to use the self-test properly while others had low self-testing self-efficacy, confirming similar misgivings found in other studies about accurately using the test [39, 44]. In South Africa, these concerns were validated as lack of instructions on how to interpret the self-test results contributed to a low sensitivity of the tests [45], suggesting that different strategies should be in place to ensure that potential self-testers receive additional guidance on how to accurately perform the self-test. For example, participants who received a short demonstration from a trained lay volunteer in Malawi were able to self-test with high accuracy (93.6% sensitivity, 99.9% specificity) [46]. Interventionists should consider providing additional guidance and support to participants in order to increase their self-efficacy to perform the test and thereby augmenting their trust in the result.

Our findings also revealed a low perceived willingness to seek confirmatory HIV testing and initiate treatment at the clinic among a few men due to potential stigma and discrimination. Similar concerns about stigma serving as a barrier to linkage to care among self-testers were reported in South Africa [33]. However, these concerns are not unique to self-testers as men who are diagnosed at the clinic also report stigma and threats to their masculinity and leadership role as barriers to HIV status disclosure and seeking care and treatment [47–50]. In Tanzania, masculinity norms that are associated with ideas of men being strong and dominant prevent men living with HIV from visiting the care and treatment centers [51]. Men report that going to the clinic may raise suspicion about their HIV status and potentially compromise their leadership roles [51]. In contrast, income-generating activities, and factors associated with respectable masculinity such as family and societal expectations to be a family provider and respectable role model encouraged men living with HIV to seek treatment in Uganda [49, 52]. Limited information about linkage to care for self-testing specifically among men in Sub-Saharan Africa exists [43], with one exception [53]. In Kenya, women reported that only 25% of the male partners they distributed self-test kits who had a positive self-test result sought confirmatory HIV testing and linked to care [53].

In the era of treatment as prevention, more efforts are needed to develop and implement strategies to increase linkage to care among men in sub-Saharan Africa such as the effort to develop a national campaign to address men’s fear about HIV testing and treatment in South Africa [54]. For self-testers in particular, home-based confirmatory HIV testing and treatment has been found to increase linkage to care compared to facility confirmatory HIV testing in Malawi [55]. Based on these results, future HIVST interventions in Tanzania should include a home-based confirmatory testing and treatment option to allow men to bypass seeking a confirmatory test at the clinic if they are not inclined to visit a healthcare facility. The acceptability of home-based couples counseling and testing, though different from home-based confirmatory HIV testing, has been shown to be highly acceptable in Northern Tanzania [56]. Another potential strategy to enhance linkage to care among male self-testers is the provision of food and cash assistance, which has been shown to help reduce stigma, improve livelihood, and HIV-related outcomes in Kenya [57, 58]. Similar strategies and effects of food and cash assistance were reported among men and women in Tanzania [59] and are also being evaluated in an ongoing trial [60].

The second aim of this study was to elicit recommendations directly from participants to address the negative attitudes, low perceived control and self-efficacy in the Tanzania STEP Project, a future HIVST and confirmatory HIV testing intervention targeting networks of men who socialize in camps in Tanzania. The respondents provided four suggestions and the first was to provide education about HIVST by their peers or through workshops and media in order to increase men’s knowledge about HIVST. The suggestion to first provide HIVST education is one of the reasons the English name of the study is Self-Testing Education and Promotion (STEP) [25]. Building on the peer health leadership model that our team has piloted [28] and implemented to promote HIV and gender-based violence prevention with men in Tanzania [61], a similar approach will be used in the STEP Project to recruit male camp members and train them to promote HIVST to their peers via conversation and pamphlets distribution. Since most of the participants were not familiar with HIVST, a male peer-based approach for HIVST education and promotion can improve men’s attitudes and personal agency toward HIVST and confirmatory HIV testing. In support of this approach, the Zambia Peer Educators for HIVST (ZEST) study, in which female sex worker peers were engaged to promote and distribute HIVST kits, was found to be acceptable and feasible [62, 63].

The second recommendation was to assess potential users’ readiness to respond to a positive self-test result prior to self-testing. Following this suggestion, one of the key features of the future STEP Project will be to first assess participants’ knowledge about the availability of HIV follow-up services and their willingness to follow the proper steps in case of a positive self-test result. Participants who meet this eligibility criteria will receive the modified WHO HIV testing and counseling without the risk assessment and individualized counseling. A demonstration on how to perform the self-test and interpret the different self-test result lines (negative, positive, faint negative or positive, and invalid), and information on where to seek confirmatory HIV testing and treatment will be provided to the participants. In addition, participants will be informed about the limitations of the self-test, including its inability to detect new infections during the window period [42]. Participants also recommended that mHealth strategies should be incorporated to provide post-test counseling for participants with positive self-test results. Participants mentioned that this could encourage them to seek confirmatory HIV testing and prevent HIVST-related harms. This recommendation aligns with other HIVST studies that have proposed offering participants in-person or phone-based counseling and reminders and options for linkage to care services [33, 64]. Current mHealth and virtual platforms used for online and phone-based HIVST and counseling [45, 65], facility-based HIV testing, and linkage to care and treatment [66–68] can be adapted to provide the pre-and-posttest support that self-testers will need in Tanzania. For example, a study conducted in China found that using an online video to promote HIVST with online real time instructions and pre-test/protest counseling was efficacious in increasing HIV testing in the intervention group (89.8%) compared to 50.7% in the control group [69]. In addition to providing self-testers in future HIVST interventions with mobile phone numbers for study counselors, a partnership can be formed with existing organizations such as the National AIDS Helpline organized by the Tanzania Youth Alliance Organization (TAYOA) to provide HIV counseling over the phone and information about HIV treatment services and voluntary medical male circumcision via text messages [70]. One of the goals of the STEP Project will include adding a mHealth component to provide follow-up services for self-testers.

### Limitations

Though our findings support other similar studies, we only interviewed men who are camp members and therefore the findings are not generalizable to all men in Tanzania. Secondly, we did not assess men’s ability to use and follow HIVST instructions or interpret HIVST results. Thus, the self-efficacy they described for HIVST would not necessarily be indicative of their actual self-test performance. In addition, mostly young men who reported to be HIV-negative were included, leaving out the experiences and perceptions of older men and men are living with HIV who had to seek confirmatory HIV testing and treatment. Including men living with HIV in future HIVST studies could provide further insight and support for other men who may contract HIV throughout the study. Future studies should include older men and also compare men’s perceptions toward HIVST after viewing other demonstration materials beside videos (e.g. hands-on demonstration, print materials) to determine which method is more effective for informing potential self-testers.

## Conclusion

HIVST was acceptable to this group of men recruited from camps, with the majority reporting positive attitude and high personal agency toward HIVST and confirmatory HIV testing. Negative attitudes towards HIVST can be addressed through peer-based programs that recruit and train peers to promote HIVST in their networks. In addition, assessing one’s knowledge in preparation of accessing HIV services, offering home-based counseling, and providing pre-and-post test support via mHealth technologies can increase the likelihood that self-testers will confirm their HIV status and initiate treatment. Delivering a package of services around HIVST (rather than thinking of it as a stand-alone technology) may increase uptake when the test becomes available and mitigate some of the anticipated social barriers.
